# Solutions to the fertility equation in beef embryo recipients

**DOI:** 10.1590/1984-3143-AR2024-0041

**Published:** 2024-08-12

**Authors:** Mario Binelli, Cecilia Constantino Rocha, Alexandra Bennett, Abdul Waheed, Halima Sultana, Mariângela Bueno Cordeiro Maldonado, Fernando Silveira Mesquita

**Affiliations:** 1 Department of Animal Sciences, University of Florida, Gainesville, FL, USA; 2 Institute of Continuing Education & Extension, Cholistan University of Veterinary and Animal Sciences, Bahawalpur, Punjab, Pakistan; 3 Universidade Federal do Pampa, Uruguaiana, RS, Brasil

**Keywords:** cattle, embryo transfer, uterus, pregnancy

## Abstract

In beef cattle operations that conduct embryo transfer, the overall success depends on the pregnancy outcome that results from every pregnancy opportunity. In this review, we dissected the main components that determine if a recipient will sustain the pregnancy after embryo transfer up to calving. Specifically, we describe the effect of the uterus on its ability to provide a receptive environment for embryo development. We then discuss the capacity of the embryo to thrive after transfer, and especially the contribution of the sire to embryo fitness. Finally, we review the interaction between the uterus and the embryo as an integrated unit that defines the pregnancy.

## Introduction

The pregnancy outcome to embryo transfer (ET) has a major impact on the economic sustainability of the activity. Practitioners and researchers strive to push the limits of fertility in cattle under ET. But what are the factors determining pregnancy success to ET? In this review we explore the concept of the following fertility equation:


Pregnancy outcome = effect of the uterus + effect of the embryo + effect of uterus x embryo + E (1)

The effect of the uterus is associated with the ability of the recipient to provide the adequate environment for embryo development; the effect of the embryo refers to its ability to hatch from the zona pellucida, elongate and signal the maternal reproductive tissues of its presence and to initiate placentation; the interaction of the uterus and embryo measures the ability of embryos fertilized with the same sire to develop in uteri with different characteristics, and the error term (E) refers to factors extrinsic to gestation but that may impact the pregnancy outcome. For the purpose of this review, we will focus on the fertility equation in what applies to the beef female, under ET programs, during the first 4 weeks of gestation. The effect of the embryo will be addressed by the abilities of different sires to generate embryos of contrasting developmental potential. We chose this window because it represents the period of most embryonic losses during the gestation (Reese et al., 2020). Therefore, this is the time in which positive interventions will result in the greatest impact on pregnancy. We will review each element of the fertility equation and discuss specific research advancements and impacts in the pregnancy success to ET.

### Effect of the uterus

In ET programs, the uterine function of recipients must be programmed to be receptive to the embryo at transfer and successfully drive elongation, maternal recognition of pregnancy, implantation and placentation. Optimal uterine function to receive the embryo consists in providing an adequate milieu of nutrients and growth factors in the uterine luminal fluid. Such fluid is enriched with secretions from the endometrial luminal and glandular epithelial cells, and they support embryo development during the first 3 weeks of gestation (Silva et al., 2023a; Forde et al., 2014; Bazer et al., 2008) Regulation of uterine function towards embryo receptivity is provided mainly by the sex steroids, estradiol (E2) from the dominant follicle during estrus and progesterone (P4) from the corpus luteum (CL) in the diestrus (Forde et al., 2009; Mesquita et al., 2015; Martins et al., 2022; Northrop et al., 2018). Thus, manipulations in the temporal dynamics, magnitude, and intensity of the sex steroids concentrations during pregnancy, and the estrous cycle preceding it, influence pregnancy success in ET programs (Pereira et al., 2016; Martins et al., 2022). The isolated and combined effects of E2 and P4 on uterine programming are discussed next.

Estrus is a hallmark of fertility. Embryo transfer to recipients that show estrus increases pregnancy/ET (P/ET) 3.3-fold compared with non-estrus recipients (Pereira et al., 2016). Increased pregnancy performance in cows that display estrus is likely due to the greater circulating E2 concentrations that drive uterine function to support the pregnancy. For example, recipients that showed estrus had similar P/ET on day 30 (39.7%) than recipients that did not show estrus but received an injection of 17β estradiol at the moment of receiving an ovulation-inducing injection of GnRH. In contrast, P/ET of recipients that neither showed estrus nor received an injection of 17β estradiol decreased to 27.4% (Ketchum et al., 2023). Interestingly, when the same authors evaluated P/ET by molecular markers on days 19 and 24, and by ultrasonography on day 30, the differences in pregnancy loss between non-estrus cows and the other groups (non-estrus + 17β and estrus cows) were only significant on days 24 and 30. Such results suggested that most pregnancy loss due to insufficient 17β-estradiol concentrations in recipients that did not show estrus occurred after day 19.

Although estradiol is a large contributor for pregnancy success in females that show estrus, it appears that there are other factors that are also responsible for increased pregnancy results. That is because in females that show estrus, E2 concentrations were not associated with pregnancy/AI (P/AI) up to day 16 of gestation (Northrop et al., 2018), and P/ET up to day 30 of gestation (unpublished data – Binelli lab). This is also in agreement with reports showing that in females that show estrus, the size of the dominant follicle was not associated with pregnancy by ET or AI (Perry et al., 2007; Pereira et al., 2016). Different results were observed for the area of the CL on day 7, in which there was only a weak association with P/ET when cows were induced to ovulate (Pugliesi et al., 2019) or when cows displayed estrus and ovulated naturally (unpublished data - Binelli Lab). Likewise, E2 concentrations in females that show estrus were not associated with the total concentration of uterine luminal proteins and glucose (Northrop et al., 2018). In previous work from our group, P/ET in recipient cows that displayed estrus and received 5 embryos was 68% (Martins et al., 2018). Five embryos were transferred to reduce the likelihood of pregnancy losses due to developmentally compromised embryos. An interpretation was that the pregnancy losses observed in that study were at least partially caused by the variability in uterine function among cows, despite the fact that they showed estrus behavior. Collectively, standing estrus and E2 are among the best described factors in the peri-estrus period that influence fertility in recipient females.

During diestrus, P4 concentrations program endometrial function and affect pregnancy success. For example, recipients manipulated to have increased P4 concentrations 4 days after estrus, followed by similar concentrations of P4 on the day of embryo transfer (day 7), had larger conceptus on day 16 of gestation (Clemente et al., 2009). These authors demonstrated that P4-induced effects on pregnancy success were mostly induced by changing endometrial function, rather than affecting the embryo directly. Interestingly, concentrations of P4, increased pharmacologically during early diestrus, did not change the abundance of P4 receptor (*PGR*) transcripts in luminal epithelial cells (Batista et al., 2019). In contrast, the uterine luminal fluid composition changed drastically. Increased P4 concentrations from days 3 to 14 after estrus changed the metabolomic composition of the uterine luminal fluid on days 12, 13, and 14 (Simintiras et al., 2019a, b). These metabolomic changes proved that P4 affects amino acid flux and the concentrations of metabolites important for conceptus elongation, such as arginine, fructose, glutamate, and mannitol/sorbitol. Furthermore, in a recent study using laser capture microdissection for transcriptome analysis, authors defined that most of the P4 effects on the endometrium were detected in the glandular epithelium, rather than the luminal epithelium or stroma (Pereira et al., 2022). These findings reinforce the idea that P4 impacts the uterine luminal fluid composition by regulating glandular secretion.

Progesterone concentrations are also associated positively with the expression of *FOXA2*, which is a factor that activates the differentiation from luminal epithelial to glandular cells (Pereira et al., 2022), and induces glandular hyperplasia and hypertrophy (see (Gray et al., 2001) for review). Additional evidence supports the idea that P4 is required to prime the endometrium to conceptus-induced stimuli, such as the expression of interferon-stimulated genes (ISGs), prostaglandins, and cortisol (Spencer et al., 2004; Song et al., 2007; Bazer et al., 2008; Rocha et al., 2023). Altogether, P4-induced changes in endometrial functions contribute to a favorable uterine environment for conceptus development, conceptus elongation, and consequent pregnancy success.

The fact that natural estrus display (i.e., a proxy of sufficient E2 concentrations) and physiological diestrus fluctuations of P4 only guarantees pregnancy in 50 to 60% of ET recipients in most studies, motivated research in our group to understand the integrated roles of hormonal, ovarian, and uterine variables potentially affecting receptivity to the embryo. Our interest in understanding the diversity in uterine programming in cows that show estrus started with the findings of Silva et al. (2021). In that study, heifers that displayed estrus naturally grouped into two clusters based on differential dynamics of uterine luminal fluid accumulation and endometrial thickness in the peri-estrus period (i.e. 2 days before and after estrus) and different concentrations of amino acids in the uterine luminal fluid, measured 4 days after estrus. We speculated that had these heifers been transferred, it would be likely that one of these profiles would be associated with greater pregnancy success. This is based on the fact that greater endometrial thickness 48 hours before AI, and the dynamics of amino acids concentration in the luminal fluid as early as day 7 post estrus have been associated with pregnancy success (Forde et al., 2014; Souza et al., 2011). In our subsequent work, we identified that the luminal epithelial cells transcriptome, measured on day 4 after estrus, was associated with pregnancy outcome of ET recipients (Martins et al., 2022). More importantly, gene expression from RNAseq analysis was adjusted by the concentrations of P4 on day 4, so that the association of pregnancy outcome and the transcriptome was not confounded by the effects of P4 on the uterus. It is likely that these differences were driven by stimuli that took place in the peri-estrus period and that affected the transcriptional profile, as well as the endometrial function, in early diestrus.

Our group further tested this idea in a series of studies. First, we pharmacologically manipulated the concentrations of P4 in the previous diestrus of cows, which consequently changed the size of the dominant follicle and concentrations of E2 when cows displayed estrus. The metabolomic and transcriptomic signatures were analyzed on days 4, 7, and 14 after estrus. In brief, P4 manipulation in the previous diestrus caused minimal changes in the uterine metabolome post-estrus (Silva et al., 2023a), but caused dramatic effects in the transcriptomic signature of luminal epithelial cells (Silva et al., 2023b). Lastly, we validated an approach to harvest and culture epithelial cells and associated the outcomes of in vitro treatments with in vivo responses of the cow from which the cells were harvested (Rocha et al., 2022). Using this model, we manipulated uterine programming by giving or not P4 from days 2 to 4 after estrus, or, giving or not an intravenous anti-inflammatory injection on day 4 after estrus. Luminal epithelial cells were harvested and cultured after the treatment. Briefly, manipulations in the cows affected interferon-t response and proliferation rates of cultured endometrial epithelial cells (unpublished data – Binelli lab). In vivo programming persisted after the stimuli were removed, and cells were submitted to 5-7 days of culture. These findings were considered evidence of endometrial epithelial cellular memory. Whether memory is also present in vivo still needs to be investigated, but if the hypothesis is confirmed, memory from peri-estrus stimuli would be a plausible explanation for the differential endometrial function observed in the early diestrus of cows that show estrus. Overall, beyond E2 and estrus, there are undefined stimuli taking place during the peri-estrus period which program endometrial function and drive receptivity to the embryo and, consequently, pregnancy success. The nature of such programs warrants investigation.

The uterine transcriptome may be used to predict pregnancy outcome. Binelli and collaborators retrospectively compared the endometrial transcriptome of biopsies collected on day 6 after artificial insemination between cows that succeeded or failed to maintain the pregnancy (Binelli et al., 2015). A set of nine transcripts was upregulated in pregnant cows. Among them, *FRAS1*, *DIO2*, and *PNMT* had the greatest fold-change (from 2.9 to 3.7). In a more recent study using recipient cows, a minimally invasive collection of uterine luminal cells by endometrial cytology was performed on day 4 after estrus (Martins et al., 2022). On day 7, recipients received an ET, and on day 30 they were diagnosed as pregnant or non-pregnant. By RNAseq, authors defined 25 transcripts as potential predictors of the pregnancy outcome on day 30. Among them, *SCARNA2* had the greatest area under the curve (0.82), specificity (83.3%) and sensitivity (80%). Machine learning approaches have also been used to predict the outcome of pregnancy using the endometrial transcriptome measured on day 7. A set of 50 transcripts was identified to predict pregnancy outcome, with 96.1% accuracy (Rabaglino and Kadarmideen, 2020). Another approach to investigate uterine competence and predicting pregnancy success was based on the fertility classification of animals (Moraes et al., 2018). In this study, heifers submitted to serial ET were classified as high-fertile, subfertile, or infertile according to their pregnancy outcome. When the same heifers were submitted to ET, P/ET was 4.4-fold greater on day 17 in fertile and subfertile heifers than infertile ones. A transcriptional analysis did not detect significant difference in the endometrium of nonpregnant fertile, subfertile, and infertile heifers. However, when the transcriptome of the pregnant endometrium of fertile and subfertile heifers was analyzed, extracellular matrix structure and cell adhesion were dysregulated in subfertile compared with fertile heifers. Thus, prior classification of heifers according to their fertility potential would be an alternative to predict pregnancy outcome. Collectively, these results indicate that the transcriptional signature of the endometrium from days 4-7 after estrus can be used to predict the pregnancy outcome in embryo recipients.

In summary, estrus, E2, P4, and additional hormonal, nutritional, environmental, genetic and other variables that impact the physiology of the peri-estrus period influence endometrial function and likely affect the pregnancy outcome after ET. Some of these variables are natural targets for technological interventions that aim to maximize uterine performance in ET programs. Some options from data published previously that can be used to maximize uterine performance are: (1) to perform ET only in females that show estrus, for greater pregnancy results, or transfer the most valuable embryos to cows that showed estrus (Madureira et al., 2022); (2) to optimize and increase precision in estrus detection for ET by using automated activity monitors. Preliminary data from our group has shown that onset of estrus is detected precisely using automated activity monitors (unpublished data – Binelli lab); (3) to assess the blood perfusion of the CL by color Doppler ultrasonography (Pugliesi et al., 2018). Blood perfusion of the CL is strongly correlated with P4 concentrations on day 7 (Rocha et al., 2019), an important regulator of endometrial function during diestrus. Briefly, if the blood perfusion of the CL was greater than 45% on day 7, an increase of 1.22-fold in P/ET was observed (Pugliesi et al., 2019). Altogether, there are readily applicable options available in the market that could be used to optimize results in ET programs, related to endometrial function. The economic viability of them is what still needs to be assessed for widespread implementation in commercial operations.

### Effect of the embryo

The quality of the embryo at ET is a critical determinant of pregnancy outcome. Quality of the embryo refers to its intrinsic ability to continue developing successfully after transfer into a given recipient. Regarding in vitro-produced embryos, properties from both the oocyte and the sperm cells individually affect embryo quality. For the purpose of this review, we will focus on the contributions of the sire (i.e., sperm cells) to embryo quality.

Inherent variability exists among bulls regarding fertility and subsequent pregnancy success. For example, the variation of bull fertility in artificial insemination (i.e., P/AI) ranges from 26 to 76% (Fair and Lonergan, 2018; Ortega et al., 2018; Zoca et al., 2023). Cows mated with sires classified as having high early embryonic mortality experience 3.7 greater odds of pregnancy loss between days 24 and 31 of gestation. Similarly, cows serviced with sires classified as having high late embryonic mortality experience 3.7 greater odds of pregnancy loss between days 31 and 60 of gestation (Franco et al., 2020). Based on these findings, it is reasonable to speculate that this trend will be observed in other systems that use semen, such as in vitro embryo production. Variable fertility in the field may translate to the observed variability among bulls in their ability to produce embryos, in vitro, that are capable of sustaining the pregnancy after ET. A few studies that focus on the effect of the sire used for in vitro embryo fertilization and the subsequent pregnancy maintenance. There are contrasting data both for blastocyst development and pregnancy success among sires following ET (Morotti et al., 2014; Lacerda et al., 2020).

The prominent difference observed among dairy bulls classified according to high (≥ 3.3) vs. low (≤ -5.1) sire conception rates (SCR) was the percentage of embryos that reached the blastocyst stage (35.6 and 42.6%, respectively) whereas the ability to fertilize the oocyte was similar (Ortega et al., 2018). There is evidence that earlier cleavage following fertilization results not only in greater blastocyst development but also greater blastocyst hatching rates (Ward et al., 2001). High fertility bulls were shown to have more advanced embryos with increased cell numbers compared with low fertility bulls, at the same time in culture (Eyestone and First, 1989; O’Callaghan et al., 2021). Similarly, high fertility bulls had significantly greater accessory sperm numbers around the oocyte than low fertility bulls (12.7 vs. 2.9, respectively) which positively related to embryo quality (DeJarnette et al., 1992; O’Callaghan et al., 2021). High fertility bulls also had increased conceptus survival compared to low fertility bulls (59.4% and 45.0%, respectively). Those conceptuses that did survive, however, had similar length among bull fertility groups (O’Callaghan et al., 2021). Additionally, P/ET was significantly different among sires of both *Bos taurus* and *Bos indicus* breeds, ranging from 23 to 52% (Morotti et al., 2014). Differences in bull fertility could be explained by differences in sperm variables, such as mitochondrial membrane potential. For example, lower mitochondrial membrane potential resulted in greater blastocyst rates for in vitro-produced embryos (Selvaraju et al., 2009; Siqueira et al., 2018). Similarly, lower acrosome integrity and lower motility before the use of a Percoll® gradient system were associated with greater blastocyst development (Siqueira et al., 2018).

Sires not only contribute to early embryonic development, but also to the success of placentation. Pregnancy-associated glycoproteins (PAGs) may serve as markers for placental function and late embryonic/early fetal mortality. In beef cattle, circulating concentrations of PAGs are decreased between days 25 to 41 of gestation in cows that experienced late embryonic or early fetal loss compared with females that maintained pregnancy (Perry et al., 2005; Pohler et al., 2013). Consistently, PAGs concentrations were shown to be influenced by sire (Franco, 2018). Gene ontology analyses demonstrated that pregnancies identified as having low PAGs concentrations, on days 25 and 36, had dysregulated expression of genes associated with embryonic and placental development, both in the trophectoderm and the endometrium (Melo et al., 2022). Conceptuses from different sires have been shown to have varying expression of genes (HAND1 and CSH2) in trophectoderm cells (Ortega et al., 2018). In mice, HAND1 is involved in placentation via giant cell proliferation (Scott et al., 2000),while CSH2 is a giant cell-specific gene (Ortega et al., 2018).Therefore, decreased expression of these genes may contribute to giant cell proliferation and differentiation, which are crucial for the formation of placentomes (Greenstein et al., 1958; Wooding, 1992). Such dysregulation likely explains the poor pregnancy outcome, at least partially.

In summary, there is clear evidence that the sire used for embryo production plays a major role in the pregnancy outcome. Such role includes influence in embryonic development in vitro and, after transfer, in utero. After conceptus elongation, and proper attachment, placental formation and function are largely dictated by the sire. The blood concentration of PAGs may be used as a proxy for placental quality and indirectly measure the ability of the sire to contribute to a successful gestation.

### Effect of the uterus by embryo interaction

Perhaps the most challenging element to study in the fertility equation is the interaction between the uterus and the embryo. Indeed, complex, simultaneous, mutual reprogramming of both the uterine and the embryo function is required for a successful pregnancy continuation after embryo transfer (Binelli et al., 2022). Do embryos produced from a given bull have similar capacities to develop in every recipient? Is a given recipient able to support the development of different embryos without distinction? These questions are starting to be addressed using in vitro models, but the in vivo data available is very limited. Elegant in vivo studies demonstrated the capacity of the endometrium to mount differential transcriptional responses, during the third week of pregnancy, when exposed to embryos of distinct developmental potential (i.e., artificial insemination, in vitro-produced and chromatin transfer-derived embryos) (Bauersachs et al., 2009; Mansouri-Attia et al., 2009). Such results were interpreted as evidence of endometrial sensitivity and plasticity, which allow for physiological adjustments according to the embryo’s signals and needs. Further investigation of the endometrial transcriptome comparing pregnant vs. cyclic heifers within the first two weeks of pregnancy/estrous cycle suggested that endometrial function prior to the maternal recognition of pregnancy is mostly regulated by conceptus-independent factors (Forde et al., 2011, 2012). The conceptus-independent endometrial regulation adds an extra layer of complexity to in vivo studies of embryo-endometrium communication. More recently, however, in vitro and in vivo models revealed the early signs of embryo-endometrium communication. Interestingly, co-culture of endometrial tissue explants or primary culture of bovine endometrial epithelial cells with pre-hatching embryos induced changes in the endometrial transcriptional profile, dependent (Sponchiado et al., 2020) and independent (Passaro et al., 2018) of the direct contact with the embryos. Moreover, presence of blastocysts in inseminated cows modulated the metabolomic composition of the uterine microenvironment and endometrial transcriptional profile in vivo (Sponchiado et al., 2017, 2019). Current evidence strongly supports an early establishment of embryo-endometrium communication and suggests a relevant role of the endometrial programming during the first week of pregnancy for subsequent gestation.

In agreement with embryo-dependent regulation of the endometrium prior to maternal recognition of pregnancy, several recent studies reported evidence of IFNτ-independent embryo-endometrium crosstalk around the period of pregnancy establishment. Mathew et al. (2019) not only confirmed that day 17 endometrial tissue explants differentially respond to embryos of distinct developmental potentials (i.e., AI vs. IVF-produced), but also reported embryonic sex-dependent and IFNτ-independent endometrial regulation. Further supporting the endometrial sensitivity to embryos of distinct phenotypes, O’Callaghan et al. (2021) demonstrated that embryos derived from fertility classified bulls elicited differential response of day 15 endometrial explants according to the bulls’ fertility potential (e.g., higher vs. lower fertility). The fertility phenotype model was also insightful when assessing the maternal contribution to embryo-endometrium communication. The endometrium from high fertility or subfertile heifers responded differently to in vivo exposure to embryos of similar developmental potential, whereas the different endometrial fertility phenotypes were able to induce differential regulation of the embryonic transcriptional profile (Moraes et al., 2018). These data further support the endometrium as both, a sensor of embryos with different developmental potentials, and a differential regulator of embryos with similar developmental potentials.

Limited advances in the understanding of biological mechanisms underlining embryo-endometrium crosstalk are partially justified by technical limitations and may be uncovered following technological breakthroughs. On this note, De Bem et al. (2021) recently reported for the first time the microfluidic in vitro primary culture of the two bovine endometrial cell types, epithelial cells and stromal fibroblasts. The successful establishment of the microfluidic environment, maintenance of cell viability, and observed response to glucose and insulin challenges, provided an additional level of control to study embryo-endometrial communication under in vitro conditions.

In light of empirical evidence of better reproductive fitness of certain bull-cow/heifer pairs, we propose the considering an alternative paradigm to approach unsolved questions in the field: that of cryptic female choice (CFC). CFC corresponds to mechanisms within the female reproductive tract that favor sperm cells from specific males (Kekäläinen, 2021). The female reproductive tract imposes a highly specific sperm selection process, likely at the individual spermatozoon level, which has long been interpreted as a tool to filter fertilization-incompetent sperm and avoid polyspermy (Fitzpatrick and Lüpold, 2014; Kekäläinen and Evans, 2018). In humans, it has been proposed that reproductive success could result from the genetic matchmaking orchestrated by the female reproductive tract, aiming to select compatible gamete pairs (Kekäläinen, 2021). Human Leukocyte Antigen (HLA), a member of the major histocompatibility complex (MHC), has been reported to be involved with the female sperm selection process in humans. Compatible male-female gamete pairs have dissimilar HLA patterns, therefore, greater MHC diversity potentially leads to greater immunocompetence (Penn and Potts, 1999). HLA is expressed on the sperm membrane and is present in female reproductive tract secretions (Rizzo et al., 2007; Sereshki et al., 2019). Exposure of different sperm samples to follicular fluid and cervical mucus of different females revealed that sperm performance in those fluids varies significantly according to the male-female pairs (Fitzpatrick et al., 2020; Jokiniemi et al., 2020). Bovine-human conserved traits such as potential exposure to multiple “partners”, small volume and concentrated ejaculate, intravaginal semen deposition, fibrous and tightly closed cervix and ovulation of single follicle may support exploring the Bovine Lymphocyte Antigen (BoLA) family of genes as a candidate sperm-selecting molecule.

In spite of major advances in the broad characterization of the temporal, spatial and qualitative endometrial response to juxtacrine, paracrine and endocrine stimuli during early pregnancy, we are likely only scratching the surface of the intricate embryo-endometrium crosstalk. Exploratory studies have been inspiring new mechanistic hypotheses of upstream regulators and downstream effectors that need to be confirmed as relevant players in uterine biology. Also, whether embryos from a given high fertility bull will be successful regardless of the recipient, or a high fertility recipient can rescue a suboptimal fertility embryo remains to be solidly determined. Advances in in vitro and in vivo approaches, technological resources, and cues from other species about evolutionary conserved reproductive processes are likely to be instrumental in advancing the field.

## Conclusions, perspectives

**Figure 1:**
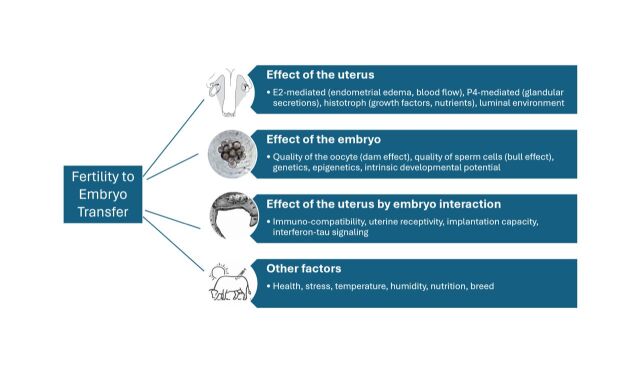
Components of the fertility equation that determine the pregnancy outcome to a single embryo transfer.

Laboratories, companies, veterinarians, and producers around the globe strive to optimize calving rates of cattle receiving ET. Efforts are usually focused to improve the performance of each determinant of reproductive performance, the receptive uterus and the competent embryo, and to understand the biology of their intricate communication (Figure 1). Progress in the field stemmed from a better understanding of conditions that favor pregnancy success, such as the choice of sires for in vitro embryo production, the hormonal conditions associated with uterine receptivity and molecular characteristics of the receptive uterus. Also, tools to predict and measure the likelihood of pregnancy success have been developed, such as panels of molecular and endocrine markers associated with the reproductive fitness of both the embryo and the recipient. There is a major knowledge gap that, however, remains to be explored: how do embryos and uteri of different competence interact in vivo to determine gestational result? What is the biochemical / genetic / immunological nature of the embryonic-uterine relationship that results in a positive pregnancy? Can it be predicted? Can it be diagnosed? Research is warranted in all aspects of the fertility equation, especially if it is approached in an integrated, multi-compartmental manner.
